# Medical gas-lighting, diagnostic odyssey and self-advocacy among women with premenstrual dysphoric disorder from nine countries

**DOI:** 10.1177/13591053251401286

**Published:** 2026-01-15

**Authors:** Meghan Mosalisa, Rizwana Roomaney

**Affiliations:** 1 Stellenbosch University, South Africa

**Keywords:** premenstrual dysphoric disorder, qualitative research, phenomenology, women’s health, medical gas-lighting

## Abstract

Premenstrual Dysphoric Disorder (PMDD) is a cyclical condition similar to premenstrual syndrome (PMS), with symptoms arising in the late luteal phase. Studies highlight barriers to care, including misdiagnosis, missed diagnosis, and limited practitioner knowledge. Most qualitative research has focussed on the USA, UK, and Australia. This study explored the healthcare experiences of 27 women from nine countries diagnosed with PMDD using a phenomenological approach. Semi-structured interviews were analysed thematically in ATLAS.ti, generating four themes: (1) medical gas-lighting and the importance of diagnosis, (2) diagnostic odyssey, (3) access to healthcare, and (4) self-advocacy. Findings reveal that negative healthcare experiences often delay diagnosis and care-seeking while fostering mistrust in healthcare systems. Experiences were similar across countries, regardless of healthcare structures. While diagnosis can be validating, its benefits may depend on available resources. Greater awareness of PMDD among healthcare providers is essential for improving patient care.

## Introduction

Premenstrual Dysphoric Disorder (PMDD) is a condition which affects menstruating women^1^ and includes both psychological and physical symptoms such as mood dysregulation, headaches, breast tenderness, anhedonia, brain fog, marked changes in appetite, sleep disturbances and decrease in energy (American Psychiatric Association, 2013; Bhatia and Bhatia, 2002; Cunningham et al., 2009; Lanza di Scalea and Pearlstein, 2017). Prevalence studies have shown that approximately 2%–8% of women may have PMDD (Cunningham et al., 2009; Roomaney and Lourens, 2020; Tschudin et al., 2010; Yonkers and Simoni, 2018). PMDD is often referred to as a more severe form of Premenstrual Syndrome (PMS), however, the two conditions differ in overall severity and impact of symptoms of women’s quality of life (Del Mar Fernández et al., 2019).

PMDD has a negative impact on patients. Studies show that women who screen positive for PMDD symptoms report lower quality of life (QOL) than their counterparts who do not screen positive (Thakrar et al., 2021; Yang et al., 2008). Studies among university students indicate that drinking alcohol (Rezende et al., 2022); having irregular menstrual cycles, severe menstrual pain; poor social support (Chekol et al., 2024); and ruminative thinking (Roomaney and Lourens, 2020) are associated with increased PMDD symptoms.

The vast majority of PMDD research is quantitative in nature, with only a handful of papers qualitatively exploring the impact of PMDD on patients. One study examined the impact of PMDD on women in their workplaces (Hardy and Hardie, 2017). Participants included 17 women from the United States, Britain and Australia (Hardy and Hardie, 2017). More recent qualitative studies have explored aspects such as diagnosis and treatment (Chan et al., 2023; Osborn et al., 2020) and the narratives of patients who were in the recovery space (Buys, 2024). Osborn et al. (2020) interviewed 17 women in the UK who reported that they were misdiagnosed, felt ridiculed and dismissed by doctors and that their diagnostic journey led to an ongoing mistrust of medical professionals. Participants also described the impact of PMDD on their lives, stating that their symptoms limited their careers, that they struggled to cope at work and that their interpersonal relationships were negatively impacted (Osborn et al., 2020). As a result, women reported secondary difficulties such as eating disorders, substance misuse, and suicidal ideations (Osborn et al., 2020). Similarly, Chan et al. (2023) interviewed 32 women who reported having PMDD, who also stated that they were misdiagnosed, experienced medical gaslighting and felt that their doctors failed to recognise symptoms of PMDD.

Medical-gaslighting refers to healthcare professionals dismissing or invalidating patients symptom experiences, potentially resulting in a decrease in health-seeking behaviours and patients second-guessing their symptom severity and experiences (Fraser, 2021; Tormoen, 2025). Participants reported that they had to advocate for themselves to be diagnosed and many self-diagnosed before being diagnosed by a medical professional (Chan et al., 2023). However, this study was methodologically limited by the fact that not all participants were diagnosed with PMDD and that majority had other comorbidities.

A recent qualitative study explored the narratives of seven participants (3 of whom were self-diagnosed) with PMDD (Buys, 2024). Participants were from Australia, America, the UK and Turkey. Again, participants reported being misdiagnosed, dismissed, gaslit and silenced by healthcare providers (Buys, 2024). Healthcare providers seemed uninformed regarding PMDD and once again participants were forced to advocate for themselves to get diagnosed (Buys, 2024). Participants also were not all diagnosed with PMDD.

While these qualitative studies provide some insights into the healthcare experiences of women with PMDD in the UK, Australia, the US, and Turkey (one participant), the experiences among patients in other geographical locations have not yet been described. Our aim was to improve on the methodology of previous studies by only including participants who were formally diagnosed with PMDD and including participants from geographical locations outside of those in previous PMDD research. Our research question was as follows:

What were the healthcare experiences of women diagnosed with PMDD globally?

## Materials and methods

### Method

The aim of this study was to qualitatively explore the lived experiences of women with PMDD from several countries. As the intent of this study was to qualitatively explore individual experiences with a disorder, descriptive phenomenology, as based on the writings of Husserl, was the most appropriate approach to facilitate this objective (Giorgi et al., 2017; Giorgi and Giorgi, 2008). In this paper, we describe one aspect of the lived experience, namely the experience of diagnosis and treatment. The approach was applied using in-depth, individual interviews with participants, which allowed them to provide a rich description of their personal experiences.

### Participants

Participants were recruited using convenience and snowball sampling methods. The inclusion criteria for this study were as follows: 18 years or older, able to communicate and understand English or Afrikaans and diagnosed with PMDD by a mental or medical health professional. Women were excluded if they did not have a clinical diagnosis of PMDD, were self-diagnosed or reported PMS symptoms not classified as PMDD.

A flyer containing the study information was posted onto Facebook PMDD support groups. The Facebook groups were selected using convenience sampling. We conducted a search on Facebook for PMDD support groups and contacted several group administrators. We obtained permission to post the study flyer on 11 international PMDD group pages.

We opted for a pragmatic approach in determining the sample size as proposed by Braun and Clarke (2019). We considered our research question, the method of data collection and analysis, depth of information and homogeneity of participants in deciding on the sample size. Our participants were homogenous in that they were living with the same condition, namely PMDD but were heterogenous because they experienced these conditions in different settings as participants were recruited globally. The recommended sample size for homogenous participants are 6–12 participants (Guest et al., 2006), whereas the recommended sample size for heterogenous participants range from 15 to 20 (Marshall et al., 2013). Malterud et al. (2016) recommends information power in determining the sample size, which also takes into account factors such as the depth of interviews and analysis in determining the sample size. We considered these factors, including the fact that the study was a descriptive phenomenological study using thematic analysis and decided that a sample of between 20 and 30 participants was appropriate. However, this was only a guide we used at the start of the study. We recruited until we were satisfied that information power was obtained.

This study involved 27 women diagnosed with PMDD. The participants in this study were recruited internationally from South Africa (*n* = 5), the United Kingdom (*n* = 3), the United States of America (*n* = 11), Romania (*n* = 1), Denmark (*n* = 2), Sweden (*n* = 1), Australia (*n* = 2), Canada (*n* = 1) and India (*n* = 1). Additional demographic information is available in Table 1.

**Table 1.** Description of participant demographics.

Demographic	Total
Age	*n*
18–25	3
26–30	3
31–35	8
35–40	4
41–45	7
46–50	2
Current relationship status	
Single	7
Committed relationship	7
Married	12
Divorced/separated	1
Children	
Yes	13
No	14
If Yes, how many?	
1	8
2	2
3	2
No. of people in household	
1	5
2	8
3	8
4	4
5	2
Current employment	
Student/employed	24
Unemployed	3

### Data collection

Data was collected using semi-structured interviews. Interview questions were based on the health-related quality of life framework developed by Ashing-Giwa (2005). The questions relating to healthcare experiences were the focus of the current paper and we asked participants to reflect on their experiences of diagnosis and treatment. Prior to the commencement of the interviews, participants were made aware that participation in the study was completely voluntary and they that may withdraw from participating at any point through the interview. This was communicated to ensure participants felt safe to communicate their honest feelings and to remove themselves from the research process where needed. Interviews were between 45 minutes and 1 hour and 45 minutes and conducted using Zoom and Microsoft Teams. Participants were given the option of keeping the cameras on or off and all provided consent to audio record the interviews. Interviews were then transcribed and entered into Atlas t.i. data management software for analysis.

### Qualitative analysis

The researchers hold a phenomenological position in exploring the subjective experiences of participants. Data analysis was guided by Braun and Clarke’s (2019), updated guidelines in using thematic analysis. Inductive and deductive coding methods were used as analysis was driven by the content of the data and theory. The authors familiarised themselves with the data by reading through transcriptions and listening to the interview recordings. Following this, initial codes were developed, edited, and grouped. This process was done jointly between authors. Finally, grouped codes were developed into four themes, one of which is the basis of this research article.

Lincoln and Guba’s (1986) four guidelines for trustworthiness were used to uphold trustworthiness in this study. An interview journal was kept for the interviewer to document her thoughts before and after each interview. This facilitated in reflexivity through the period of data collection and analysis. Additionally, authors held regular meetings to discuss the interviewing process, codes and findings. This study used phenomenology as its research approach, which required the researcher to stay in a reflective state because it is believed to be a crucial step in the research process (Eberle, 2013; Usher and Jackson, 2017). Therefore, continuous introspection and self-awareness were required as it was essential for the researchers to remain objective and conscious of their own prejudices and preconceived notions regarding PMDD and quality of life during data collection and analysis.

While the researchers are female and have experience with menstruation, they did not have experience with PMDD. However, they found that as women they could relate to some aspects of participants’ experiences. In interpreting the data, they tried to centre participants’ experiences and reflect on what the experience must have been like for participants. The researchers had a profound sense of empathy and respect for participants. As a result, using a phenomenological approach enhanced the researchers’ capacity to recognise and accurately convey the experiences of women with PMDD.

### Ethical considerations

This study received ethical approval from the Health Research Ethics Committee (HREC) at Stellenbosch University (S21/05/095). All participants read and signed the consent forms before participating in the study. Participants names were anonymised in the writing of study results, all names used in this paper are participants pseudonyms. As the topic was sensitive in nature, we made free counselling available to participants if they were to become distressed during or after the interview.

## Results

This study sample consisted of 27 women aged between 19 and 50 (mean age 32). As evident in Table 1, most women did not have children (*n* = 14), were either full-time students or employed (*n* = 24) and were between the ages of 31 and 35 (*n* = 6).

For the purposes of this article, healthcare refers to interactions between patients, doctors, and other medical and mental healthcare practitioners.

Four themes were identified that described participants’ healthcare experiences. The themes were: “Medical gaslighting and the importance of a diagnosis,” “Diagnostic Odyssey,” “Access to healthcare,” and “Advocating for myself.”

### Medical gas-lighting and the importance of a diagnosis

The term medical gas-lighting was presented by a participant and refers to the invalidation of patient experiences by healthcare professionals. Even though other participants may not have explicitly mention the term, they alluded to it when they reported dismissive attitudes of doctors towards their severe premenstrual symptoms, doctors not acknowledging PMDD as a valid diagnosis and doctors disregarding patient’s experiences by attributing their severe premenstrual symptoms to PMS. Participants stated that medical gas-lighting made them feel unimportant, dismissed, and often lead to delayed health-seeking behaviours and PMDD diagnosis as women were reluctant to seek help because of medical gas-lighting. For some participants, medical gas-lighting was interpreted as a lack of support from their healthcare systems.

*“. . .but practitioners that do not believe that this is a real thing because it’s not very, you know, it’s not visible [. . .] Medical gas-lighting is a really good term here. That’s hard, that’s really hard.”* (Kayla, United States of America)

*“. . .my PMS symptoms are quite hectic. And his [doctor] response was you don’t have to react to those feelings. And he was saying it in a very dismissive [way], like. Just take control of, of, of your life sort of way. Um, yeah, just deal with it sort of way. And that feeling sticks with me. Like you [doctor] don’t understand, and obviously you [doctor] don’t believe that these hormones have an effect that is so severe that we feel that we need help or we, we cannot control it by ourselves anymore.”* (Liezl, South Africa)

In both the above quotes, the women illustrated how they were medically gaslit in their respective healthcare systems. The healthcare systems in America and South Africa differ substantially, with an under-resourced and overburdened system in South Africa in comparison with a more accessible system in America. Both women described having their experiences be dismissed by their healthcare providers and explained that their experiences of dismissal were demotivating and had an overall negative impact on their mental well-being. Additionally, in the quote from Liezl, she described how her healthcare practitioner appeared to explain to her that her symptoms of PMDD were something she could control, which the participant interpreted as unempathetic and dismissive of her symptom experience. Furthermore, these quotes demonstrate that treatment of severe premenstrual symptoms may have been similar throughout healthcare systems, irrespective of participants geographical location. This may allude to a systemic problem regarding the chronic dismissal of women’s health concerns within healthcare structures.

Participants reported that receiving a PMDD diagnosis validated their difficult healthcare experiences and served as an acknowledgement of their struggles with their severe premenstrual symptoms. In addition, they reported that a diagnosis was an essential step towards receiving treatment for their severe premenstrual symptoms and was interpreted as an acknowledgement and validation of their experiences. As a result, a PMDD diagnosis was viewed as an essential part of participants PMDD journey.

*“I just felt validated. Like everything, it [diagnosis] allowed me to look back at my life and actually makes sense of a lot of life changes that I’ve made that I was very impulsive about. And it kind of, yeah, it [diagnosis] helped us [family] make sense of everything. And there was also a feeling of being cared for, really. That someone did care, that I mattered. That there were people out there who were looking into this illness [PMDD]. And I only found out about it [PMDD] six months before, so it was all really new to me. I was learning so much. And I cried a lot. There was pure relief, really, that actually, I wasn’t going crazy.”* (Micaela, United Kingdom)

In this quote, Micaela described how important her PMDD diagnosis was as it allowed for better understanding of herself, her past experiences and current PMDD symptoms. As a result, receiving a PMDD diagnosis played an important role in supporting her psychological well-being. Her PMDD diagnosis may be seen as a catalyst for her healing and self-acceptance. These sentiments were expressed by most participants, where women not only highlighted the importance of being diagnosed for healthcare reasons but also described it benefitting their mental health and self-concepts. Furthermore, the value placed on being understood and receiving validation appeared to have led to Micaela feeling supported by her healthcare providers.

Another participant reported feeling relief when she received her diagnosis:

*I was super, super relieved. To be honest. Cause I didn’t know what was wrong. No one could treat it. No one was helping me. Now that I had a name I could actually go and see OK fine, this is it. Read more about it, the more I read about it the more it made sense to me. So I was actually very, I felt just relieved.* (Maya, South Africa)

Maya’s relief when she was diagnosed was related to her making sense of her symptoms. The diagnosis empowered her as she was able to find out more about her condition and may have signalled the start of her recovery journey. Similar to the quote by Micaela, Maya’s quote illustrates the importance of a diagnosis in positively impacting on women’s psychological well-being and in better understanding their disorder and symptoms.

However, a diagnosis was not helpful for all participants, as some expressed that treatment was not effective. For example, in the following quotation, a participant from India indicated that the diagnosis was not helpful because of her geographical and social contexts.

*I don’t really think that the diagnosis really. . . changed anything because there wasn’t really. I mean, being in India also being in two other countries as well. I mean I never really received a whole lot of support. It was really me managing it all my life so.* (Prajna, India).

In the quotation above, the participant reported that her life did not change when she was diagnosed with PMDD as no support was made available to her. Additionally, Prajna alludes to her social contexts being the reason for her not receiving the support she required to her assist her in understanding and managing her diagnosis of PMDD. Therefore, in the instance for Prajna, her diagnosis did not add value or make a significant change to how her disorder impacted on her life or psychological wellbeing as she was unable to receive the necessary support and guidance because of her social context. An explanation for this could be socio-cultural factors, such as stigmatisation and marginalisation of women within different populations. Thus, in addition to effective treatment, psychosocial support is an important factor in relation to well-being of patients with PMDD.

### Diagnostic odyssey

In their pursuit of a diagnosis, participants found that healthcare practitioners were not sufficiently knowledgeable about PMDD to diagnose or treat their severe premenstrual symptoms. As a result, participants stated that a lack of practitioner knowledge negatively impacted on the quality of care they received. This meant that participants had to consult several healthcare professionals before receiving the PMDD diagnosis. Moreover, the process of consulting multiple doctors for participants’ symptoms were reported to negatively impact on their psychological well-being. This process is best described as diagnosis shopping. It is important to note that diagnosis shopping was reported by many participants from several geographical location, potentially demonstrating the universality of this issue. This quote illustrates how one participant, from Romania, had to consult with three healthcare practitioners to receive a diagnosis.

*“Ever since I’ve been going to the gynaecologist and I’ve been asking them [gynaecologist] if they [gynaecologist] know about it [PMDD], one of them didn’t [know about] it, even after I told her the Romanian word, she just wrote something else because she didn’t even have the notion. [. . .] a psychiatrist, one month ago, who told me, PMDD is not a real diagnosis. And so I told her, but it is in the DMS-5. . . she [psychiatrist] told me, “I [psychiatrist] haven’t really read the DSM since it was in the third edition. . .”* (Adina, Romania)

In this quote, Adina described both her struggles in seeking professional help for her severe premenstrual symptoms and the lack of practitioner knowledge regarding PMDD within her healthcare context. Additionally, in this quote Adina described how her psychiatrist dismissed her symptoms because the psychiatrist had no knowledge of PMDD prior to her consultation with Adina. This may illustrate the gap in practitioner knowledge in identifying and understanding premenstrual disorders in women. This quote illustrates her interactions with her psychiatrist and illustrates why many women in this study reported consulting several professionals for a diagnosis. The psychiatrist admission that they had not read the DSM since the third edition is disturbing and indicates complacency and disregards for the DSM. The lack of knowledge regarding PMDD from a psychiatrist is alarming and resulted in poor patient care.

Another participant, from South Africa, also described her experience seeking a diagnosis in the following quotation:

*I feel like, ugh, to be very honest people really don’t know anything about PMDD. . .. Because this, I had psychologist hopped, psychiatrist hopped so much. And when I got, yeah, the education gap is . . . massive. Like, people just don’t know about it. They don’t know how to treat it. They treat it like normal depression.* (Maya, South Africa)

Maya also described the impact of the knowledge gap regarding PMDD among psychologists and psychiatrists that she consulted and expressed her dissatisfaction with misdiagnosis and lack of practitioner knowledge related to treatment. Later in the interview, she described multiple misdiagnosis including major depressive disorder and burnout. Both Adina and Maya experienced diagnosis shopping and misdiagnosis in different geographical locations and contexts, potentially illustrating the global issue of lack of knowledge in diagnosing and identifying premenstrual disorders and symptoms. Although both countries have different healthcare systems, the barriers in care which women experienced were similar. These experiences may also be shaped by social attitudes towards women and women’s health in general.

The search for a diagnosis had a negative impact on their psychological well-being. Participants reported seeking feelings of frustration and demotivation, which negatively impacted their health-seeking behaviours. This is illustrated in the following quote, where a participant described how, after a negative experience with her healthcare practitioner, she stopped seeking help for her severe premenstrual symptoms. Lucinda was later diagnosed by a nurse practitioner.

*“So when I first talked to that doctor, the one that I mentioned who just was like here, I can give you an antidepressant and that’s pretty much it. I felt like that was very cold and clinical and I felt like it didn’t. . .it wasn’t the support I felt like I needed at the time [. . .] So I felt like it just felt so transactional, like transactional medicine, and that really deterred me from seeking uhm clinical support again for a long time.”* (Lucinda, United States of America)

### Challenges in accessing healthcare

Participants reported multiple factors associated with challenges in accessing appropriate healthcare to support their PMDD symptoms. Challenges described by participants as barriers to accessing healthcare were doctors’ location, financial costs, medical aid scheme (medical insurance) rules and healthcare practitioner expertise. For some, lack of accessibility was related to healthcare practitioners being out of reach by distance and an inability to travel for healthcare consultations. Furthermore, the financial burden of ineffective treatments, diagnosis shopping, consulting with practitioners, travel costs and access to health services proved for some participants to be too much. This led to participants paying for treatments out of pocket, causing further financial burden. These barriers are described in the following quotes:

*“So I live in a pretty small town. It’s like 40,000 people and we’re surrounded by mountains and ocean, so we don’t have a lot of options. . . So I did not pursue a different psychiatrist because I really don’t have any other options here without spending a lot of money.”* (Bethany, United States of America)

*“It was extremely expensive to see these practitioners I mean the psychiatrist was like R3500 (185 Euro) per consultation, the gynaecologist was easily around R1000 or R2000 (53 to 106 Euro). So you know it’s really expensive*, (Penelope, South Africa)

*“Trust me, like I had to fight and fight and fight, for over two years to find a gynaecologist (who understood PMDD). . . It’s horrible. So right now, I cannot even find a psychiatrist to help me, because there is none that is covered by my healthcare. And right now, I cannot afford it and I need a psychiatrist, and I haven’t been able to find one because the ones who specialize in PMDD like cost $500 per session.”* (Grace, United States of America)

In the first quote, Bethany described that she could not afford seeking healthcare support outside of where she lived because of financial burden. She described that doctors closest to her were not knowledgeable about PMDD and premenstrual disorders to appropriately treat her. This illustrates that lack of practitioner knowledge may have presented itself as an additional barrier to accessing appropriate healthcare. Similarly, Grace described that financial barriers to seeking support, such as her medical scheme not covering her psychiatrist visits. Therefore, because of financial burden, Grace had to stop attending therapy and taking her medication. While Bethany and Grace were based in America, similar sentiments were expressed by participants in other countries. This may illustrate the universality of challenges in accessing healthcare for women with premenstrual complaints, irrespective of healthcare system and geographical location. In the second quote, Penelope from South Africa, mentioned the costs of seeking healthcare within the private sector in South Africa. These costs are not within reach for most South Africans.

### Advocating for myself

As previously mentioned, participants stated that medical gas-lighting played a role in their delayed diagnosis. Participants stated needing to search for a diagnosis by consulting multiple health professionals in order to have their symptoms acknowledged or finally receive a diagnosis of PMDD. Many participants reported that they had to advocate for themselves to get a diagnosis and appropriate treatment for their PMDD and specific symptoms. Several participants reported conducting their own information searches and then informing healthcare practitioners of their suspicions that they had PMDD. In the quote below, Quinn described how she brought the potential diagnosis of PMDD to her general practitioner to receive a clinical diagnosis of the disorder.

*“I searched again. I was like, something has got to be here, like I’ve got to be able to find what’s wrong with me. And that’s where I just stumbled across something about PMDD and I’ve, you know, I’d looked, every, I don’t know, probably few months, few years, I’m not sure, for, you know, answers, and then I never found anything and no doctors ever said anything about that. That could be related to my hormones or anything. So I basically self-diagnosed and then went to the doctor.”* (Quinn, Denmark)

Due to years of misdiagnosis and frustrating experiences with unsupportive healthcare practitioners, Quinn stated that she had given up on searching for an answer for her severe premenstrual symptoms. Ultimately, after her own research and finding PMDD, Quinn consulted with a doctor who agreed on the diagnosis of PMDD. This quote demonstrates the importance of self-education in participants attempts at receiving recognition for their symptoms and a potential PMDD diagnosis. It is also concerning that participants had to suggest a diagnosis of PMDD and once again indicates a knowledge gap among professionals. Additionally, this quote illustrates the urgency with which the participant searched for a diagnosis, further demonstrating the importance of a diagnosis to women struggling with severe premenstrual complaints.

While seeking treatment and medical support, participants stated the need to advocate for themselves within their healthcare systems. Participants advocated for themselves by requesting referrals to specialists as doctors did not think their symptoms and experiences warranted a specialist referral. Moreover, participants stated that they needed to provide evidence for their severe premenstrual symptoms to gain appropriate recognition by healthcare practitioners. Therefore, participants reported having to track their cycles and symptoms and research available treatments for PMDD to better advocate for themselves during healthcare consultations. Upon receiving treatment, some participants reported dissatisfaction with the effectivity of their treatment leading to prompt discussions with their healthcare providers regarding possible treatment modifications. Participants sought their own information on treatment and some suggested treatment options to their doctors. These options included hysterectomy, oophorectomy and chemical menopause. However, these treatments were scarcely offered to women as an option and doctors were reported to be apprehensive in offering more aggressive treatments. Participants stated that their practitioners’ reservations were not appropriately discussed with them and that there appeared to be a discrepancy between patient and practitioner goals. This is demonstrated in the quote below:

*“Because I’m really trying to improve my life, my quality of life, and I’m like. What are my options? And I said to him. So can we put me in chemical uhm, menopause? And he said no. And you still have lots of, you know, he was very about the sexual active side. [. . .] I was upset. I was sad. I felt demotivated again because I just can’t come to the I just can’t settle that this is the way I’m going to [live] and it’s only just gonna get worse. That’s what he said.”* (Vanessa, Denmark)

In this quote the participant’s experience illustrates how the treatment goals of the doctor were trumped by the needs of the patient. We interpreted this as a barrier between patient and practitioner understanding which resulted in a break in trust that further lessened the patients belied in the doctor’s ability to appropriately advocate for them. Furthermore, this break in trust could negatively impact on patients’ psychological well-being, potentially leading to despondency towards being diagnosed with PMDD. This participant was not helped with an alternative treatment method.

## Discussion

In this paper we describe the healthcare experiences of 27 patients who were diagnosed with PMDD. We sought to improve on the methods used in other qualitative PMDD studies, by including participants from countries where this topic has not yet been explored and by only including patients with an official PMDD diagnosis. Participants reported medical gas-lightning and described the importance of a diagnosis. They also discussed having to consult with several healthcare professionals before receiving a diagnosis. Participants described the challenges that they experienced in accessing healthcare and finally a need to advocate for themselves. While several of these aspects have been reported in qualitative studies (Buys, 2024; Chan et al., 2023; Osborn et al., 2020), we report more nuanced findings based on the location of participants. The findings are not only aligned to PMDD studies, but similar to other women’s health conditions such as endometriosis and PCOS, indicating that patients seeking treatment for women’s health conditions may be treated similarly.

Overall, participants reported negative healthcare experiences, which were often rooted in medical gas-lighting. This finding was reported among participants in different countries, indicating that it may be a universal phenomenon among patients seeking a diagnosis for PMDD. While other studies also reported medical gas-lighting in relation to PMDD health-seeking behaviour (Buys, 2024; Chan et al., 2023), we added to this by including quotes from participants in countries such as South Africa. Medical gas-lighting was identified by Chan et al. (2023) as a provider barrier to diagnosis of PMDD. However, medical gas-lighting is not only described in relation to PMDD but is commonly reported in other women’s health conditions such as polycystic ovarian syndrome (PCOS) and endometriosis (Ismayilova and Yaya, 2022; Mikesell and Bontempo, 2022; Soucie et al., 2021). Among women with PCOS, the chronic dismissal of symptoms by healthcare providers were also identified as a barrier to diagnosis, resulting in a delayed diagnosis (Ismayilova and Yaya, 2022). Medical gas-lighting in the current context could be related to limited knowledge about PMDD or an attitude that doctors may have towards women, which may see their symptoms as malingering and therefore find it easy to dismiss.

Even though it was challenging for participants in the current study to obtain a diagnosis, most spoke about the importance of a diagnosis. A diagnosis was seen as validating and provided some relief to participants who saw it as essential for treatment and recovery. Similar sentiments were expressed in PMDD studies among participants in the US (Chan et al., 2023) and the UK (Osborn et al., 2020). However, a participant in the current study from India did not experience her diagnosis as helpful, as it did not improve her condition and she was not offered any support. This may indicate different experiences among patients in different countries and may be related to the availability of resources. This finding warrants further investigation about PMDD diagnosis in low- and middle-income countries.

Participants reported having to consult several healthcare practitioners before being diagnosed. We refer to this as diagnosis shopping and while this was mentioned in other PMDD studies (Chan et al., 2023; Osborn et al., 2020), it is not exclusive to PMDD. In a study conducted by Ismayilova and Yaya (2022), 41% of participants reported consulting three or more doctors before being diagnosed with PCOS. Furthermore, diagnosis shopping occurred irrespective of participants geographical location as women from different countries explained similar experiences when accessing their countries healthcare services for their symptoms.

Several primary obstacles to healthcare access were reported by participants. These included the location of healthcare practitioners, financial costs, regulations of medical aid programmes, and practitioner proficiency with PMDD. Research on women’s health in general in the US (Carsdoso et al., 2021) and among American participants with PMDD (Chan et al., 2023), identified financial difficulties, health insurance, and healthcare providers expertise as potential barriers to receiving appropriate healthcare. According to Chan et al. (2023), a major obstacle to patient care for women with self-reported PMDD in the United States was a lack of health practitioner knowledge on PMDD. A possible explanation for this is described by Paul and Pal (2024) explaining a possible gap in the training of doctors in identifying and diagnosing premenstrual disorders. Additionally, Paul and Pal (2024) state research trends of PMS and PMDD show that PMDD literature is present more within psychiatric research, and not as present in medical journals. These findings may suggest a reason for the healthcare practitioners’ gap in understanding and diagnosing premenstrual disorders. A lack of access to adequate healthcare for premenstrual disorders could lead to women discontinuing treatment due to financial restrictions or delaying health-seeking behaviours, which was demonstrated by the participants of this study. We found that the challenges to accessing healthcare extended beyond the US and illustrated this by sharing information from a South African participant who reported the high cost of private healthcare. It is important to note that within the healthcare sector in South Africa, patients can opt for costly private treatment (either funded through medical aid plans or cash) or for less costly or even free services at public healthcare facilities. Patients at private facilities are able to choose their doctors, while patients at public facilities have to see a doctor on duty and often do not get to see the same doctor. Barriers to care may differ in different context and more descriptive studies of these experiences are needed.

Participants of this study included women from nine countries, showcasing the healthcare experiences of women with PMDD from a global perspective. Refer to Supplemental Appendix A for information on the different healthcare systems. Additionally, women in this study reported that stigma surrounding menstruation and related disorders impacted on their health-seeking behaviours. Upon experiencing severe premenstrual symptoms, women reported feeling reluctant to access healthcare support as a result of this stigma. According to a systematic review by Saxena et al. (2023), factors influencing women’s access to healthcare in Asian and African countries were economic status, social norms, community support, personal knowledge and beliefs and economic status. Looking specifically at knowledge and beliefs as a barrier to accessing healthcare, women’s myths and misconceptions regarding disease. These are similar to the findings presented in this study by participants in lower-middle-income countries (South Africa and India). Participants healthcare experiences were reported in a similar way across countries when it came to experiences associated with patient care, stigma associated with premenstrual complaints, diagnosis shopping and medical gas-lighting. Research assessing women’s healthcare experiences and conducted in the United States of America and Ireland found similar findings (Cardoso et al., 2021; Windrim et al., 2024). In both the USA and Ireland healthcare practitioners invalidated patient gynaecological experiences and women were forced to fight for care to gain recognition for their symptoms (Cardoso et al., 2021; Windrim et al., 2024).

Although religion and spiritual beliefs were not mentioned as a barrier for women in this study, the findings described by Saxena et al. (2023) support the notion that health-seeking behaviours are impacted by cultural factors, similar to the societal stigma’s described by participants in this study. Moreover, societal stigma surrounding menstruating women were also reported to negatively impact health-seeking behaviours and women’s reluctance to report severe premenstrual complaints. This is similar to the sentiments expressed in Greenhalgh (2022) article, describing how women’s experiences of consistent misdiagnosis and dismissal of symptoms can be attributed to gender-based bias within the healthcare system. Furthermore, stigma regarding mental illness and women’s health were described in several studies as a barrier to care (Ismayilova and Yaya, 2022; Mikesell and Bontempo, 2022; Osborn et al., 2020). Participants in this study reported needing to advocate for themselves within their healthcare systems to receive a diagnosis. They advocated for themselves by being proactive to get a diagnosis, conducting their own research on PMDD, suggesting to healthcare practitioners that they had PMDD, tracking the menstrual cycles and making treatment recommendations, Participants reported that medical gas-lighting, healthcare practitioners trivialising patients’ severe premenstrual symptoms and ineffective treatments were reasons why participants felt it necessary to advocate for themselves within their healthcare systems. These findings are similar to existing women’s health research among patients with endometriosis and PCOS (Hantsoo et al., 2022; Ismayilova and Yaya, 2023; Mikesell and Bontempo, 2022; Wren and Mercer, 2022). Studies assessing women’s health and healthcare experiences described self-advocacy and persistence as common practices in women’s attempts to receive a diagnosis, accessing specific treatments and specialists and combating dismissive healthcare practitioners (Hantsoo et al., 2022; Ismayilova and Yaya, 2023; Mikesell and Bontempo, 2022; Wren and Mercer, 2022). According to Wren and Mercer (2022), women highlighted the importance of persistency in attempting to have their concerns and symptoms acknowledged by healthcare practitioners and in attempting to access medical specialists in efforts to receive a diagnosis for their endometriosis-related symptoms. Similarly, Mikesell and Bontempo (2022) indicated that women often needed to self-advocate within their healthcare systems by suggesting endometriosis as a diagnosis, proposing alternative treatments, and attempting to access medical specialists knowledgeable in endometriosis.

### Conclusion

This study has both strengths and limitations. This study sheds light on the complexities faced by women who live with a disorder that is not properly acknowledged by medical professionals and is invisible to those who do not experience it. Furthermore, this research offers vital insights into the experiences of women in their healthcare systems.

The first limitation pertained to language, as interviews could only be conducted in two languages as these were spoken by the interviewer and these was no funding for translation. Another limitation related to the countries in which participants resided. Even though this study builds on previous research by including participants from several countries, it is still only limited to these nine countries. A third limitation is that our use of convenience sampling through social media may have introduced selection bias. Responses from participants were largely negative and it may have been that those who volunteered for the study did so due to their negative experiences, whereas those who had positive experiences may not have felt a need to participate. The final limitation was that participants often found it difficult to describe their experiences relating to how socio-ecological factors impacted on their experiences with PMDD. When this occurred, participants would pause, reflect on their experiences and answer as best as they could. In addition, interviews were a suitable method of data collection and allowed for participants to share their broad experiences.

The findings presented in this paper illustrate the difficulties that patients with PMDD navigate within the healthcare system. By including participants from several countries, we were able to demonstrate similarities and differences in these experiences. Further research should be conducted globally, focussing on the experiences of patients with PMDD, especially in low- and middle-income countries.

## Supplemental Material

sj-docx-1-hpq-10.1177_13591053251401286 – Supplemental material for Medical gas-lighting, diagnostic odyssey and self-advocacy among women with premenstrual dysphoric disorder from nine countries Supplemental material, sj-docx-1-hpq-10.1177_13591053251401286 for Medical gas-lighting, diagnostic odyssey and self-advocacy among women with premenstrual dysphoric disorder from nine countries by Meghan Mosalisa and Rizwana Roomaney in Journal of Health Psychology

## Notes

1. For the purposes of inclusivity, the term “women” is used in this paper as this commonly used in the literature. We acknowledged that people who menstruate may not identify as women and that the term ’people who menstruate’ is more inclusive as it represents cisgender women, non-binary and trans individuals.
