# Supplementary material for: Medical gas-lighting, diagnostic odyssey and self-advocacy among women with premenstrual dysphoric disorder from nine countries
Source: J Health Psychol. 2026 Jan 15;31(8):3227–40. doi: 10.1177/13591053251401286 (PMC13287426; doi:10.1177/13591053251401286)
Supplement: sj-docx-1-hpq-10.1177_13591053251401286 – Supplemental material for Medical gas-lighting, diagnostic odyssey and self-advocacy among women with premenstrual dysphoric disorder from nine countries [file sj-docx-1-hpq-10.1177_13591053251401286.docx]

**Supplementary material**

**Appendix A: Summary of Healthcare sectors in each country**

| **Country** | **Public Sector** | **Private Sector** | **Access** | **Insurance System** |  |
| --- | --- | --- | --- | --- | --- |
| South Africa | Overburdened | High quality | Unequal, income-based | Private medical schemes, planned NHI | |
| United Kingdom | Dominant (NHS) | Supplementary | Universal | Optional private insurance | |
| United States | Limited | Dominant | Insurance-dependent | Private (ACA, Medicare, Medicaid) | |
| Denmark | Strong | Limited | Universal | Mostly public, minimal private use | |
| Romania | Universal (in theory) | Growing | Regional/income disparities | Public mandatory, growing private | |
| Sweden | Strong | Complementary | Universal | Optional private insurance | |
| Australia | Strong (Medicare) | Strong | Universal with private incentives | Public + optional private | |
| Canada | Strong | Supplementary | Universal | Private insurance for non-covered care | |
| India | Underfunded | Major provider | Unequal, improving | Growing public and private insurance | |

*This table is AI generated*
